# Investigation of Sexes and Fertility Potential of Female Russian Sturgeon (*Acipenser gueldenstaedtii*) and Male American Paddlefish (*Polyodon spathula*) Hybrids

**DOI:** 10.3390/life14070818

**Published:** 2024-06-27

**Authors:** Katalin Bogár, Jelena Stanivuk, Aliz Géczi, Georgina Lea Fazekas, Balázs Kovács, Bence Lázár, Mariann Molnár, László Ardó, Uroš Ljubobratović, Gyula Kovács, Dániel Péter, Eszter Várkonyi, Jenő Káldy

**Affiliations:** 1 Research Centre for Fisheries and Aquaculture, Institute of Aquaculture and Environmental Safety, Hungarian University of Agriculture and Life Sciences, H-5540 Szarvas, Hungary; bogar.katalin@uni-mate.hu (K.B.); stanivuk.jelena@uni-mate.hu (J.S.); geczi.aliz@uni-mate.hu (A.G.); fazekas.georgina.lea@uni-mate.hu (G.L.F.); ardo.laszlo@uni-mate.hu (L.A.); ljubobratovic.uros@uni-mate.hu (U.L.); kovacs.gyula@uni-mate.hu (G.K.); 2 PhD School of Animal Biotechnology and Animal Science, Hungarian University of Agriculture and Life Sciences, H-2100 Gödöllő, Hungary; molnar.mariann@nbgk.hu; 3 Department of Molecular Ecology, Institute of Aquaculture and Environmental Safety, Hungarian University of Agriculture and Life Sciences, H-2100 Gödöllő, Hungary; kovacs.balazs@uni-mate.hu (B.K.); peter.daniel.2@phd.uni-mate.hu (D.P.); 4 Institute for Farm Animal Gene Conservation, National Centre for Biodiversity and Gene Conservation, H-2100 Gödöllő, Hungary; lazar.bence@nbgk.hu (B.L.); varkonyi.eszter@nbgk.hu (E.V.); 5 Animal Biotechnology Department, Institute of Genetics and Biotechnology, Hungarian University of Agriculture and Life Sciences, H-2100 Gödöllő, Hungary

**Keywords:** ploidy level, gonad, testis, spermatogonia, interfamilial hybrids

## Abstract

In the present study, 10 allotriploid (3nALT) and 10 allopentaploid (5nALP) six-month-old hybrid fish and two 3nALT and four 5nALP 40-month-old hybrid fish, which resulted by crossing female Russian sturgeon *Acipenser gueldenstaedtii* (Brandt and Ratzeberg, 1833) and male American paddlefish *Polyodon spathula* (Walbaum, 1792), were investigated. It was revealed that six-month-old 3nALT and 5nALP hybrids initially had “undifferentiated” gonads, while in the 40-month-old hybrids, only testes were observed in one case of 3nALT and one case of 5nALP hybrids. The testis of 3nALT hybrids was partially developed with spermatogonia, while the testis of one 5nALP hybrid was in the second developmental stage with low spermatogonia density. We could not determine gonad differentiation in any of the cases when the hybrid individuals had the W sex chromosome. We concluded that the gonad differentiation of these interfamilial hybrids follows a similar pattern to interspecific hybrids of different ploidy parent species of the family *Acipenseridae*, which is consistent with the classical Haldane’s rule. However, it cannot be excluded that the testis of this/these hybrid(s) may produce fertile sperm after sexual maturity, depending on additional genetic, hormonal and environmental factors, and further research is required for its evaluation.

## 1. Introduction

Hybridization and polyploid formation are widespread occurrences among diverse fish species [1], and differ between taxa, thus facilitating their evolutionary processes [2]. Polyploids are divided into allopolyploids, which have a combination of chromosomes from two or more different species, and autopolyploids, which have several sets of chromosomes, but from one taxon [3]. Allopolyploids are grouped into segmental allopolyploids and genomic allopolyploids. Segmental allopolyploids are partially cross-fertile and have a similar genome, while genomic allopolyploids are cross-sterile and contain two different genomes at their time of origin [4].

The order Acipenseriformes comprises two families, *Acipenseridae* and *Polyodontidae* [5]. The *Acipenseridae* family includes 25 living sturgeon species [5], and the *Polyodontidae* family has only one extant species, the American paddlefish [6]. Hybridization is still a common event in the *Acipenseridae* family [7] because the polyploid state as well as the karyotypic and genetic similarity of sturgeons facilitate interspecific hybridization [8]. The evolution of Acipenseriformes involves more/multiple whole-genome duplication (WGD) events, which resulted in evolutionary tetraploid (4n~120), octoploid (8n~240) and dodecaploid (12n~360) karyotyped species [9]. The diploid karyotype of the common ancestor of Acipenseriformes had 2n = 60 chromosomes [10]. The first WGD occurred approximately 254.7 [11]—200 Mya (million years ago) [12], followed by at least two—five more lineage and species-specific genome duplication events in sturgeons [13]. According to recent research, the ancient common WGD of Acipenseriformes occurred at the Permian–Triassic boundary, followed by lineage-specific re-diploidization [11]. However, there is a hypothesis suggesting that sturgeon polyploidy was the original cause of hybridization events, while paddlefish polyploidy has an autopolyploid origin [14]. The unique properties of sturgeons, such as their tolerance to genetic regulatory processes, polyploidy, and the ZZ/ZW sex chromosome system, enable the development of fertile interspecific hybrids [15]. The *Acipenseridae* family is believed to have a ZZ/ZW female heterogametic sex-determination system, and the female-specific W gene loci have already been identified in four tetraploids and two octoploid species [16]. Paddlefish are also considered to have the ZZ/ZW female heterogametic sex-determination system [17]. In interspecific hybridization, if the ploidy level of the parent species is the same, the hybrids will have the same ploidy level as their parent species. In contrast, if the parent species have different ploidy levels, their hybrids will have an intermediate ploidy level between those of their parent species [18]. However, besides interspecific hybridization in the Acipenseriformes species, the male American paddlefish can also hybridize with the female Russian sturgeon [19], pallid sturgeon (*Scaphirhynchus albus*) [20] and sterlet (*Acipenser ruthenus*) [21]. In hybridization analysis, it is recommended to use the functional scale instead of the evolutionary scale, considering the re-diploidization of the sturgeon genome [18]. Based on this principle, the n~120 evolutionary tetraploid species are functionally diploids, the species with n~240 chromosomes are functionally tetraploids and the n~360 evolutionary dodecaploid species are functionally hexaploids [22]. The hybrids resulting from the cross-breeding of the American paddlefish male with the Russian sturgeon female are functionally triploid (3n~180 chromosomes) or functionally pentaploid (5n~300 chromosomes) karyotypes [19]. Functionally diploid (2n~120) karyotypes resulted from hybridizing male American paddlefish with female pallid sturgeon [20] and sterlet [21]. Sex differentiation encompasses the differentiation of the gametes into testes or ovaries [23]. Interspecific hybridization can lead to high mortality rates and a wide range of abnormalities in the development of gonads [24,25]. In animals and plants, F1 hybrids can be viable but infertile or have low fertility [24]. Triploid offspring of polyploid parents can produce viable n, 2n and 3n gametes [25]. However, according to published data, hybrids of *Acipenseridae* species with the same ploidy are mainly fertile [26]. In contrast with this, Linhartová et al., 2018 [27] concluded that in hybrids of female Russian sturgeon and male sterlet or female Siberian sturgeon and male sterlet, the development of the ovaries was interrupted, and the testes continued to develop, so limited male fertility cannot be ruled out. This study aimed to investigate the sex identification and early gonad differentiation in the evolutionary triploid (functionally diploid) 3nALT and in the evolutionary pentaploid (functionally triploid) 5nALP hybrid offsprings of the female Russian sturgeon and male American paddlefish cross.

## 2. Materials and Methods

### 2.1. Experimental Design

In the experiment, hybrid fish from two different age classes (six and 40 months old) and two ploidy levels (allotriploid [3nALT] and allopentaploid [5nALT]) were analysed. The ploidy levels were determined by counting the number of dorsal and ventral scutes according to Káldy et al. (2020) [19]. From the six-month-old fish, 10 3nALT, ten 5nALP and 10 Russian sturgeon individuals were analysed, while two 3nALT, four 5nALP and two Russian sturgeon individuals were analysed from the 40-month-old hybrid fish by genetic sex marker, gonad histology and microsatellite markers. Parental individuals of the two age classes of hybrids are not identical.

### 2.2. Reproduction and Rearing

The purebred parental fish individuals originated from stocks that have been raised for three generations or more in captivity at the Gene Bank of HAKI (Hungarian University of Agriculture and Life Sciences, Research Centre for Fisheries and Aquaculture, Szarvas, Hungary). The six-month-old fish were bred in January 2022. Eggs from one Russian sturgeon female and sperm from two American paddlefish males were used for reproduction. The breeders were injected with LHRH (luteinizing hormone-releasing hormone) analogue Des-Gly^10^(D-Ala^6^) LHRH-ethylamide (Sigma Aldrich, Burlington, MA, USA) (40 µg/kg for females and 20 µg/kg for males) to induce ovulation and spermiation. Sperm was stripped 48 h post-injection, while eggs were stripped 31–41 h post-injection using the Podushka method (first step: opening temporary stretch; second step: scalpel inserted into the stretched gonopore carefully; third step: one to two cm incision made through the dorsal area of the oviductal wall; fourth step: the eggs flow in through the oviduct and out of the gonopore), cited by Štěch et al. (1999) [28]. Immediately before fertilization, sperm was diluted with water at a ratio of 1:200 [19], and two mL of this diluted solution was added to the egg samples weighing 300 g, according to the common aquaculture practice. The water temperature for hatching was 13.0 ± 1.0 degrees Celsius. Hatching occurred six to seven days after fertilization. Then the offspring were placed in an aquarium with a volume of 120 litres. Exogenous nutrition started eight to nine days after hatching. The fish were fed with Artemia ad libitum from the start of feeding until the age of 14 days, and then a mixture of frozen Chironomus spp. and rearing fish feeds (Aller Infa Ex 0.1 mm; crude protein: 64%; crude fat: 12%; Aller Aqua A/S, Christiansfeld, Denmark) were used. At the age of three weeks, fish were fed with starter fish feed (Aller Infa Ex Granulate 0.1–0.4 mm; crude protein: 64%; crude fat: 12%; Aller Aqua A/S, Christiansfeld, Denmark), and from the age of four weeks, they were fed with rearing fish feed (Aller Infa Ex 0.4 mm; crude protein: 64%; crude fat: 12%; Aller Aqua A/S, Christiansfeld, Denmark) only. At that time, the feed dose was five percent of the body weight. At the age of four weeks, after acclimatization, the fish were transferred to a one cubic metre fish-rearing tank with a recirculation system, and feeding occurred every two hours. The water temperature in this pool was 20 ± 2.0 degrees Celsius. During the rearing, the value of dissolved oxygen of the rearing water varied between 6.5 and 8.5 mg/L, while the value of ammonia varied 0.15 ± 0.03 mg/L. The 40-month-old American paddlefish male and Russian sturgeon female hybrid fish were bred in 2019 based on the method of Káldy et al., 2020 [19]. The fish were reared up to the age of 12 months in rearing ponds with a surface area of 100 square metres. Between the ages of 13 and 40 months, we transferred the fish to ponds with a surface area of 500 square metres. In rearing ponds, the value of dissolved oxygen was varied between 4.5 and 6.5, while the value of ammonia varied between 0.2 and 0.3 mg/L. The fish were fed with Aller Aqua sturgeon rearing food (Aller Futura Ex Granulate 0.5–2.0 mm; crude protein: 58 to 60%; crude fat: 15 to 17%; Aller Bronze 3.0–4.5; crude protein: 45%; crude fat: 15%; Aller Aqua A/S, Christiansfeld, Denmark) according to their size in the amount corresponding to the water temperature, one to five percent of the body weight.

### 2.3. Determination of Ploidy

The determination of ploidy level was based on the number of dorsal and ventral scutes according to the study of Káldy et al. (2020) [19]. The number of dorsal and ventral scutes of hybrids is related to their ploidy levels (3nALT or 5nALP) and shown by relevant differences between these hybrid groups [19]. Two meristic characteristics should be considered together as the number of dorsal and ventral scutes to identify the ploidy. 5nALP was defined as a hybrid with a minimum of five ventral scutes in one row and a minimum of 12 dorsal scutes. 3nALT is a hybrid with zero to seven ventral scutes in one row and a maximum of 11 dorsal scutes. Comparing these meristic characteristics of hybrids with the maternal species (Russian sturgeon), they are lower in 3nALT hybrids, intermediate in 5nALP hybrids and higher in Russian sturgeon [19].

### 2.4. Microsatellite Marker Analysis

Fin clips were collected from the male American paddlefish and female Russian sturgeon parents, as well as from ten 5nALP and ten 3nALT six-month-old hybrid offspring and two 3nALT and four 5nALP 40-month-old hybrid individuals. To determine the hybrid origin, the allele peters from the hybrid were compared with that of the parent fish. Four pairs of microsatellite primers were used for genotyping purposes [19,29,30]. Genomic DNA was extracted according to the standard protocol of E.Z.N.A Tissue Kit (Omega Bio-tek Inc., Norcross, GA, USA). The forward primers were tailed by a 5′ 17 bp long sequence to provide “universal” oligos for a fluorescent dye-labelled attachment site (fluorescent dyes: VIC, NED and PET). One tail-specific oligo was added to each reaction mixture. The reactions consisted of 100 ng genomic DNA, 10× Dream Taq PCR buffer (it contains 20 mM MgCl_2_) (Thermo Fisher Scientific, Waltham, MA, USA), 0.4 μL dNTP (0.2 mM of each), 0.5 μL forward and reverse primer (10 μM), 0.5 μL fluorescent dye-labelled tail specific oligo (5′dye-ATTACCGCGGCTGCTGG-3′) (10 μM), and 1.0 U DreamTaq DNA polymerase (Thermo Fisher Scientific, Waltham, MA, USA) in a total volume of 20 μL.

PCR conditions included an annealing temperature (Table 1) for 30 s, initial denaturing at 94 °C for 2 min, 35 cycles of 94 °C denaturing for 30 s, extension at 72 °C for 1 min. The labelled PCR products were separated by capillary electrophoresis using ABI Prism 3500 Genetic Analyzer (Thermo Fisher Scientific, Waltham, MA, USA). The length of these fragments was determined by GeneMapper 6.0 Software (Thermo Fisher Scientific, Waltham, MA, USA).

### 2.5. Sex Determination with SSM4 Analysis

DNA isolation from the fin clips was performed using the E.Z.N.A.^®^ Tissue DNA Kit (Omega Bio-tek, Inc., Norcross, GA, USA) following the producer’s instructions. A female-specific marker SSM4 was used for genetic sex determination, which was amplified by PCR reaction [31]. The primer sequences of the SSM4 marker were (5′-CCCTGATCCTAATGTTTTCGGTTGG-3′ and 5′-AGATCACGTAGGACTTTAATCGTT-3′). Mitochondrial DNA (MT) inner reference primers were also used in the same reaction to ensure the reliability of the PCR amplification system [31]. The final volume of the reactions was 10 µL thereof, 1 µL of 10X DreamTaq Green Buffer (Thermo Fisher Scientific, Waltham, MA, USA), 0.2 µL of dNTP (10 mM, Thermo Fisher Scientific, Waltham, MA, USA), 5 mM of SSM4 primers, 1 mM of MT primers, 0.5 unit of DreamTaq Green DNA polymerase (Thermo Fisher Scientific, Waltham, MA, USA), 100 ng µL of template DNA and 6.5 µL of nuclease-free water. The first step of the PCR (Kyratec SuperCycler Trinity Revision 2.0.0, Fisherbiotec, Wembley, Australia) was a 5 min denaturation at 94 °C, followed by 35 cycles of 30 s, at 94 °C, 30 s, at 56 °C and 20 s, at 72 °C, and a final extension for 7 min. at 72 °C [31]. PCR products were inspected on 1.5% agarose gel in TBE buffer for 40 min at 70 V. Based on the size and pattern of the amplified fragments, we determined the genetic sex of each individual.

### 2.6. Histological Analysis of the Gonads

Fish were anaesthetized with 2-phenoxyethanol, and after grading their total body length and individual body mass, they were sacrificed. The gonadal particles from all three morphological regions (anterior-, mid- and posterior regions) were fixated after dissection in Bouin’s solution for 48 h using the method by Hurvitz et al. (2007) [32]. After dehydration with a series of ethanol (concentrations 96%), the tissue was cleared with xylene, and the fractions were embedded in paraffin and cut into 5 μm thick slices using Microtome (Leica Biosystems Rm2245 Semi-Automated Rotary Microtome, Deer Park, IL, USA). The slides with sections were stained with hematoxylin and eosin. The samples were examined under a light microscope (BX51, Olympus, Tokyo, Japan 40#), and their images were taken (ImageFocusAlpha 1.3.7.5177, Euromex, Arnhem, The Netherlands, 10#). The gonadal cells were evaluated and rated for the stage of development based on the terminology developed by Vizziano-Cantonnet et al. (2016) [33].

### 2.7. Statistical Analysis

Given the lack of degree of freedom and sample size, the non-parametric Mann–Whitney U test (0.05) was applied for the analyses of six-month-old juvenile fish for body weight, dorsal and ventral scutes (SPSS 29.0.1.0) (IBM SPSS Statistics, Armonk, NY, USA). For the 40-month-old fish, a 95% confidence interval was applied. Scatter plots were made to graphically present differences in ploidy, body weight and two different scatter numbers (XLSTAT).

## 3. Results

### 3.1. Hybrid Origin and Ploidy Levels of Each Hybrid Specimen

The ploidy level and differences in body weight of the twenty-six investigated hybrid individuals are displayed in Table 2.

The microsatellite marker analysis confirmed the hybridization of each offspring (Table 3). One paternal allele inherited Psp-28, Psp-32 and Spl-101 loci and two paternal alleles inherited Psp-29 loci in all hybrid individuals.

### 3.2. Genetic Sex of Hybrids and Purebred Specimens

**Figure 1:**
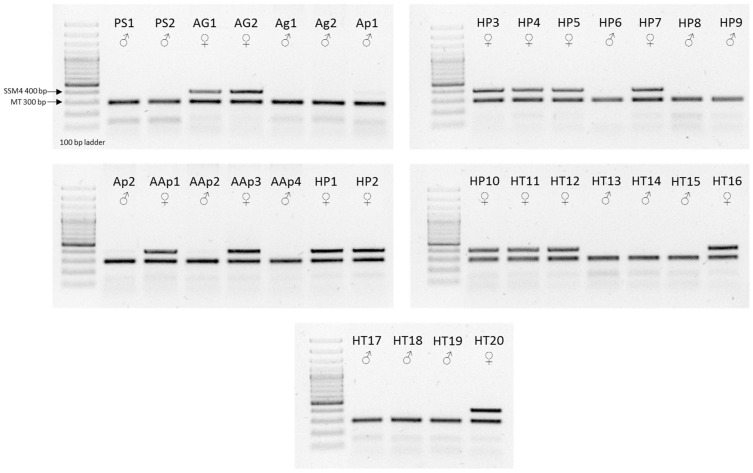
Sex determination by SSM4 female-specific DNA sequence of purebred parent species and hybrid individuals on agarose gel. Symbols: ♀ female; ♂ male.

The sex of purebred Russian sturgeon and hybrid individuals can be determined using the *Acipenseridae* sex-specific marker SSM4. Among the 6-month-old 3nALT hybrid fish, six individuals did not carry the Russian sturgeon-derived maternal allele, indicating the male (HT13–HT15 and HT17–HT19) genotype and four were carrying the female-specific sequence (HT11, HT12, HT16 and HT20) showing the female genotype. In the six-month-old 5nALP hybrid group, seven fish were identified as females (HP1-HP5, HP7 and HP9) and three fish (HP6, HP8 and HP9) were identified as males (Figure 1). The 40-month-old 3nALT hybrids (Ap1 and Ap2) were determined to be male, while two of the 5nALP hybrids were male (AAp2 and AAp4) and two were female (AAp1 and AAp2).

In Figure 1, 400 bp (base pair) shows the size of PCR products with the SSM4 female-specific DNA marker, and 300 bp shows the size of PCR products with the mitochondrial (MT) DNA fragments. PS1 and PS2: American paddlefish parent species individuals; AG1 and AG2: Russian sturgeon parent species specimens. Ag1 and Ag2: 40-month-old Russian sturgeon control individuals; Ap1 and Ap2: 40-month-old triploid (3nALT) hybrids; AAp1-AAp4: 40-month-old pentaploid (5nALP) hybrids; HP1-HP10: six-month-old pentaploid (5nALP) hybrids; HT11-HT20: six-month-old triploid (3nALT) hybrids. 5nALP hybrid: male American paddlefish (*Polyodon spathula*) and female Russian sturgeon (*Acipenser gueldenstaedtii*) pentaploid hybrid; 3nALT hybrid: male American paddlefish (*Polyodon spathula*) and female Russian sturgeon (*Acipenser gueldenstaedtii*) triploid hybrid.

### 3.3. Gonads and Gametes of Hybrids and Purebred Specimens

Among the thirty examined 6-month-old individuals, ten 3nALT hybrids (H11–H20), ten 5nALP hybrids (H1–H10) and ten control purebred Russian sturgeon revealed “undifferentiated” gonads. The ten control purebred 6-month-old Russian sturgeons had the same body mass as the hybrids.

**Figure 2:**
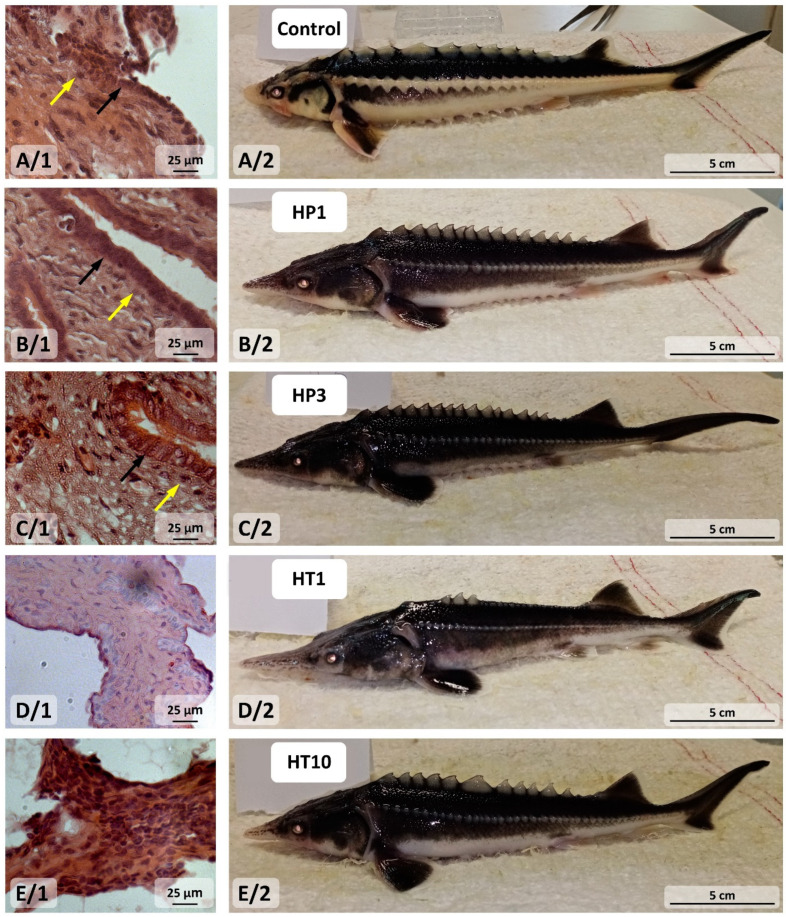
Microscopic view of an undifferentiated gonad (**A/1**) and macroscopic view of whole-body size (**A/2**) of six-month-old purebred Russian sturgeon. Microscopic view of undifferentiated gonad (**B/1**,**C/1**) and macroscopic view of whole-body size (**B/2**,**C/2**) of six-month-old 5nALP hybrids of male American paddlefish and female Russian sturgeon. Microscopic view of undifferentiated gonad (**D/1**,**E/1**) and macroscopic view of whole-body size (**D/2**,**E/2**) of six-month-old 3nALT hybrids of male American paddlefish and female Russian sturgeon. Black arrows: cells in the epithelium that have become columnar cells; yellow arrows: primordial germ cells (PGC) on the periphery of the gonad. 5nALP hybrid: male American paddlefish (*Polyodon spathula*) and female Russian sturgeon (*Acipenser gueldenstaedtii*) pentaploid hybrid; 3nALT hybrid: male American paddlefish (*Polyodon spathula*) and female Russian sturgeon (*Acipenser gueldenstaedtii*) triploid hybrid.

One control purebred Russian sturgeon (A/1) and two 5nALP individuals (B/1 and C/1) exhibited distinguishable features; specifically, the somatic cells of the epithelium, which had become columnar cells, as well as the primordial germ cells that remained at the periphery of the gonad could be identified. The gonads of two 3nALT hybrids were also “undifferentiated” (D/1 and E/1) (Figure 2).

In the case of one 40-month-old purebred Russian sturgeon individual, the detected testes were in the second development stage, as pre-meiotic seminiferous tubules with normal spermatogonia density (AG2). We could not identify gonad tissue in the other purebred Russian sturgeons.

Among the 40-month-old 3nALT hybrids, a partially developed testis with spermatogonia could be determined in one fish (Ap1). However, we found undefined gonads in the other 3nALT hybrid fish (Ap2).

**Figure 3:**
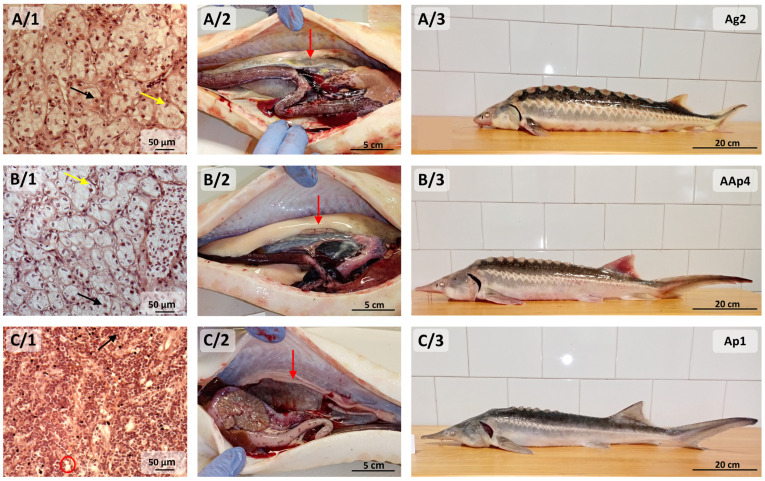
Microscopic view of pre-meiotic seminiferous tubules with normal spermatogonia density of 40-month-old purebred Russian sturgeon (**A/1**). Macroscopic view of 40-month-old purebred Russian sturgeon gonad (**A/2**) and whole-body size of purebred Russian sturgeon (**A/3**). Microscopic view of pre-meiotic seminiferous tubules with low spermatogonia density of 40-month-old 5nALP hybrid of male American paddlefish and female Russian sturgeon (**B/1**). Macroscopic view of gonad (**B/2**) and whole-body size (**B/3**) of 40-month-old 5nALP hybrid of male American paddlefish and female Russian sturgeon. Microscopic view of the partially developed gonad (**C/1**) of 40-month-old 3nALT hybrid of male American paddlefish and female Russian sturgeon. Macroscopic view of gonad (**C/2**) and whole-body size (**C/3**) of 40-month-old 3nALT hybrid of male American paddlefish and female Russian sturgeon. Black arrows: spermatogonia; yellow arrows: Sertoli cell; red arrows: gonad; red ellipse: exhibiting spaces without germ cells. 5nALP hybrid: male American paddlefish (*Polyodon spathula*) and female Russian sturgeon (*Acipenser gueldenstaedtii*) pentaploid hybrid; 3nALT hybrid: male American paddlefish (*Polyodon spathula*) and female Russian sturgeon (*Acipenser gueldenstaedtii*) triploid hybrid.

Among the four 40-month-old 5nALP hybrids, one case of fat tissue (AAp3) and two cases of undefined gonads were found (AAp1 and AAp2). For one pentaploid individual (AAp4), the gonad was well developed, and this fish specimen was found to have pre-meiotic seminiferous tubules with low spermatogonia density in the second development stage (Figure 3).

## 4. Discussion

The development of the testis occurs in six stages: Stage 1: Differentiation of testis: the testis is thread-like, and the clusters of primary spermatocytes are located in the gonad. Macroscopic sex determination at this stage is not easy. Stage 2: Proliferation of spermatogonia: spermatogonia undergo mitotic proliferation, surrounded by Sertoli cells and enclosed in cysts. Stage 3: Early spermatogenesis: the testis contains primary and secondary spermatocytes in cysts. Stage 4: Mid-spermatogenesis: the testis is well developed, and spermatocytes, spermatids and spermatozoa can be observed. Stage 5: Late Spermatogenesis: sperm predominate in the testes, and the number of spermatocytes decreases. Stage 6: Degeneration stage: the Sertoli cells are enlarged, and the sperm count is high [34,35,36]. In the evaluation of Siberian sturgeons (*Acipenser baeri*), sex differentiation could not be carried out based on morphological changes in the gonad between three to six months of age [33]. Morphologically undifferentiated gonads have even been observed in nine-month-old juvenile Russian sturgeon individuals [37]. The fertility of the male hybrids of parents with different ploidy levels has already been proven in the case of sterlet and kaluga (*Huso dauricus*) hybrids, as these hybrids were crossed with females of the parental species, and viable offspring were hatched [26]. A partially developed testis with spermatogonia was identified in the case of four- to six-year-old hybrids of sturgeon parent species differing in ploidy [27]. Our investigation revealed no differentiated gonads in the six-month-old 3nALT and 5nALP hybrid fish, whereas a partially developed testis with spermatogonia was observed in 40-month-old 3nALT fish, and in the case of a 5nALP individual, the testis was in the second stage of development, just like the identical Russian sturgeon of the same age; however, the spermatogonia density of the hybrid species was low.

The molecular mechanisms governing sex development and differentiation in sturgeons remain poorly understood [34]. Additionally, the effect of polyploidization on the genetic sex-determination system is largely unknown [15]. The ZZ/ZW female heterogametic sex-determination system has been identified in the *Acipenseridae* species [16,38,39,40] and paddlefish [17]. Male sex differentiation is a complex process in sturgeons, involving the action of male hormones and factors, along with the suppression of female genes [35]. Female sex differentiation depends on the activation of a female sex-specific gene cascade and estrogen [36]. Allopolyploid genomes behave as diploids in a biological sense [2]. In species using the ZZ/ZW sex-determination system, the presence or absence of the W chromosome determines the sex, with the number of W chromosome(s) being irrelevant [1]. However, in some cases, the dosage of sex chromosomes is important for the formation of sex [40]. In polyploid hybrids with triploid ZZW genotypes, it can result in intersex phenotypes the lower ratio of W-specific, determining genes [41]. According to the dominant W chromosome principle, the male 3nALT hybrids have a ZZZ, while the male 5nALP hybrids have a ZZZZZ genotype. If the dominant W chromosome is present in the genotype of a hybrid species, hybrids will be female individuals [42].

In the hybrids of parents with different ploidy levels in the *Acipenseridae* family, only aborted, apoptotic cells were identified in ovaries, and with a high rate, thus suggesting that they are presumably also sterile [27]. In cases with no detectable gonad, we believe to have identified the tissue that fills part of the ovary, but this must be confirmed in further studies. In contrast, fertility hybrid females were observed, which were the result of crosses with the different ploidy sterlet and kaluga [43].

In the case of some *Acipenseridae* species, the most ancient sexual determination system of vertebrates was described with undifferentiated sex chromosomes [16]. However, a question arises as to why only a partially developed testis was observed in 3nALT hybrids, whereas in 5nALP hybrids, we identified a testis at the same stage of development as the Russian sturgeon, but with low spermatogonia density. The reason for this could be either the limited number of examined individuals or, in the case of 3nALT hybrids, the ploidy of the hybrids. In 5nALP hybrids, theoretically, some gene has four alleles of Russian sturgeon origin (because of the evolutionarily ancient genome duplication and the biased polyploidization during the hybridization) and one of American paddlefish origin, whereas 3nALT hybrids have one allele of American paddlefish origin and two alleles of Russian sturgeon origin (evolutionarily ancient genome duplication). It can be assumed that in 5nALP hybrids, due to the multiple alleles from one species, one allele from the other species causes minor disturbances.

The present study proved that sex differentiation is possible in both 3nALT hybrids and 5nALP hybrids and the testis in 5nALP hybrids undergoes similar development as the testis of the Russian sturgeon of the same species with similar age and weight but with low spermatogonia density. This would also be consistent with the classical Haldane’s rule [44], according to which the homogametic sex can be fertile, while the heterogametic sex is sterile or absent.

However, the experimental design and the methods of the present study did not allow us to investigate the effect of dosage of Z and W sex chromosomes and the ratio of maternal and paternal Z sex chromosomes for gonad development in different ploidy level hybrids.

## 5. Conclusions

In this study, the gonad development of hybrid species derived from the male American paddlefish and the female Russian sturgeon was investigated for the first time. A partially developed testis with spermatogonia was identified in one 40-month-old 3nALT hybrid. The testis of one of the four 40-month-old 5nALP hybrids was found to be in the second stage of development, similar to the Russian sturgeon of the same age and body mass. However, the testis of the 40-month-old 5nALP hybrid exhibited a low spermatogonia density. It is assumed that the sex-determination system of these interspecific hybrids follows a similar pattern to the already established pattern for interspecific hybrids originating from parents of different ploidy levels within the *Acipenseridae* family. Although we only used a small number of samples, based on our preliminary results, the process likely occurs at both triploid and pentaploid ploidy levels in the hybrids. Therefore, it is not yet possible to convincingly state whether fertile sperm cells can develop in the testis, but the presence of fertile or semi-fertile male hybrid individuals cannot be ruled out.

The present results show that although the phylogeny of the American paddlefish and the Russian sturgeon diverged 180.4 million years ago, the genetic process of sex formation is so conserved that the hybrid of these two species also exhibits sex differentiation. However, whether the triploid or pentaploid testis produces fertile sperm can only be determined after the individuals reach sexual maturity.

## Figures and Tables

**Table 1:** Sequence and annealing temperature of applied microsatellite primers.

Locus	Inheritance		Primer’s Sequences (5′-3′)	Anneling Temperature (°C)	Repeat Motif
	*P. spathula*	*A. gueldenstaedtii*			
Psp-28	Disomic	Tetrasomic	F: Tail-CAAAGGCATCCCCTACCAC	56	GA
			R: GCTGGACAAAAAGTATGGAGTGC		
Psp-29	Tetrasomic	Disomic	F: Tail-GGGGTCTAATAAAATCCACCGTTC	56	GCAC
			R: TTGCCTTGTGCTCTGTGTTCC		
Psp-32	Monomorf	Tetrasomic	F: Tail-AATGACTCAGTTGTGTGCTGC	60	GT
			R: AAGTGTAGGGGAATCTCACCAG		
Spl-101	Monomorf	Tetrasomic	F: Tail-CCCTCCACTGGAAATTTGA C	52	TCTA
			R: GCAATCAACAAG GTCTCTTTCA		
Tail (17bp)	-	-	ATTACCGCGGCTGCTGG	-	-

National Center for Biotechnology Information (NCBI) accession number of locus: Psp-28: AF406738.1; Psp-29: AF406739.1; Psp-32: AF406740.1; Spl-101: AF276170.1.

**Table 2:** The ploidy level, the body weight and the number of ventral and dorsal scutes of 3nALT and 5nALP hybrid (male American paddlefish (*Polyodon spathula*) × female Russian sturgeon (*Acipenser gueldenstaedtii*) individuals.

Ploidy	Body Weight (g)	Number of Ventral Scutes	Number of Dorsal Scutes
p (Mann–Whitney)	0.000	0.000	0.002
Pentaploid (H1–H10)	61.55 ± 12.19	6.3 ± 0.9	14.6 ± 1.5
Triploid (H11–H20)	32.57 ± 10.35	2 ± 2.3	5.3 ± 3.7
Asymptotic significance 95%	0.335	0.06	0.046
Pentaploid (AAp1–AAp4)	2587.7 ± 965.21	7 ± 0.81	12.25 ± 0.5
Triploid (Ap1 and Ap2)	1930.2 ± 862.95	3.5 ± 2.12	6 ± 0.0

Abbreviations: H1-H10: six-month-old pentaploid (5nALP) hybrids; H11-H20: six-month-old triploid (3nALT) hybrids; AAp1-AAp4: 40-month-old pentaploid (5nALP) hybrids and Ap1 and Ap2: 40-month-old triploid (3nALT) hybrids.

**Table 3:** Examples of microsatellite genotypes of six-month-old 5nALP (H1–H10) and 3nALT (H11–H20) and 40-month-old 3nALT (Ap1 and Ap2) and 5nALP (AAp1–AAp4) hybrid individuals. 5nALP hybrid: male American paddlefish (*Polyodon spathula*) and female Russian sturgeon (*Acipenser gueldenstaedtii*) pentaploid hybrid; 3nALT hybrid: male American paddlefish (*Polyodon spathula*) and female Russian sturgeon (*Acipenser gueldenstaedtii*) triploid hybrid.

Individuals	Psp-28				Psp-29			Psp-32					Spl-101				
Russian sturgeon	220	222			201			134	162	164	166		304	322	330	334	
American paddlefish			*260*	*267*		*211*	*225*					*196*					*274*
HP1	220	222	*260*		201	*211*	*225*	134	162		166	*196*	304	322	330	334	*274*
HP2	220			*267*	201	*211*	*225*	134		164	166	*196*	304	322	330	334	*274*
HP3	220	222		*267*	201	*211*	*225*	134	162	164		*196*	304	322	330	334	*274*
HP4	220	222	*260*		201	*211*	*225*	134		164	166	*196*	304		330	334	*274*
HP5	220		*260*		201	*211*	*225*	134		164		*196*	304	322	330	334	*274*
HP6	220	222		*267*	201	*211*	*225*	164		164	166	*196*	304	322	330	334	*274*
HP7	220			*267*	201	*211*	*225*	134	162	164		*196*		322	330	334	*274*
HP8	220	222		*267*	201	*211*	*225*		162		166	*196*	304			334	*274*
HP9	220	222	*260*		201	*211*	*225*	134		164	166	*196*	304	322	330	334	*274*
HP10	220	222	*260*		201	*211*	*225*	134	162	164		*196*	304	322	330	334	*274*
HT11	220	222		*267*	201	*211*	*225*	134		164	166	*196*	304		330		*274*
HT12	220	222	*260*		201	*211*	*225*	134		164		*196*	304	322			*274*
HT13	220	222	*260*		201	*211*	*225*		162	164		*196*	304		330	334	*274*
HT14	220	222	*260*		201	*211*	*225*	134	162		166	*196*	304		330	334	*274*
HT15	220	222	*260*		201	*211*	*225*	134		164		*196*	304	322			*274*
HT16	220	222	*260*		201	*211*	*225*	134		164		*196*	304	322	330		*274*
HT17	220	222	*260*		201	*211*	*225*	134	162			*196*	304	322	330		*274*
HT18	220		*260*		201	*211*	*225*		162	164		*196*	304		330		*274*
HT19	220	222	*260*		201	*211*	*225*	134	162	164		*196*	304	322	330	334	*274*
HT20	220	222		*267*	201	*211*	*225*		162	164		*196*	304	322	330	334	*274*
Ap1	220			*267*	201	*211*	*225*	134	162			*196*	304	322			*274*
Ap2	220		*260*		201	*211*	*225*			164	166	*196*	304	322			*274*
AAp1	220		*260*		201	*211*	*225*	134	162	164		*196*	304	322			*274*
AAp2	220		*260*		201	*211*	*225*	134	162		166	*196*	304		330		*274*
AAp3	220			*267*	201	*211*	*225*		162		166	*196*	304	322			*274*
AAp4	220		*260*		201	*211*	*225*	134		164	166	*196*	304	322			*274*

Abbreviations: Psp-28 is disomic, and Psp-29 is tetrasomic, in paddlefish; Psp-29 is disomic, Psp-28, Psp-32 and Spl-101 are tetrasomic in Russian sturgeon (paternal alleles are labelled in italics).

## Data Availability

The data presented in this study are available on request from the corresponding author.

## References

[1] Gregory T.R., Mable K.B., Gregory T.R. (2005). Polyploidy in Animals. The Evolution of the Genome.

[2] Collares-Pereira M.J., Matos I., Morgado-Santos M., Coelho M.M. (2013). Natural Pathways towards Polyploidy in Animals: The *Squalius alburnoides* Fish Complex as a Model System to Study Genome Size and Genome Reorganization in Polyploids. Cytogenet. Genome Res..

[3] Hu F., Fan J., Qin Q., Huo Y., Wang Y., Wu C., Liu Q., Li W., Chen X., Cao L. (2019). The Sterility of Allotriploid Fish and Fertility of Female Autotriploid Fish. Front. Genet..

[4] Thompson J.D., Lumaret R. (1992). The evolutionary dynamics of polyploid plants: Origins, establishment and persistence. Trends Ecol. Evol..

[5] Peng Z., Ludwig A., Wang D., Diogo R., Wei Q., He S. (2007). Age and biogeography of major clades in sturgeons and paddlefishes (Pisces: Acipenseriformes). Mol. Phylogenet. Evol..

[6] Zhang H., Jarić I., Roberts D.L., He Y., Du H., Wu J., Wang C., Wei Q. (2020). Extinction of one of the world’s largest freshwater fishes: Lessons for conserving the endangered Yangtze fauna. Sci. Total Environ..

[7] Fontana F., Tagliavini J., Congiu L. (2001). Sturgeon genetics and cytogenetics: Recent advancements and perspectives. Genetica.

[8] Birstein V.J., Hanner R., DeSalle R. (1997). Phylogeny of the Acipenseriformes: Cytogenetic and molecular approaches. Environ. Biol. Fishes.

[9] Birstein V.J., Vasil’ev V.P. (1987). Tetraploid-octoploid relationships and karyological evolution in the order *Acipenseriformes* (Pisces). Karyotypes, nucleoli, and nucleolus-organizer regions in four acipenserid species. Genetica.

[10] Ludwig A., Belfiore M.N., Pitra C., Svirsky V., Jenneckens I. (2001). Genome Duplication Events and Functional Reduction of Ploidy Levels in Sturgeon (*Acipenser*, *Huso* and *Scaphirhynchus*). Genetics.

[11] Redmond A.K., Casey D., Gundappa M.K., Macqueen D.J., McLysaght A. (2023). Independent rediploidization masks shared whole genome duplication in the sturgeon-paddlefish ancestor. Nat. Commun..

[12] Havelka M., Kašpar V., Hulák H., Flajšhans M. (2011). Sturgeon genetics and cytogenetics: A review related to ploidy levels and interspecific hybridization. Folia Zool..

[13] Lebeda I., Ráb P., Majtánová Z., Flajšhans M. (2020). Artifcial whole genome duplication in paleopolyploid sturgeons yields highest documented chromosome number in vertebrates. Sci. Rep..

[14] Vasil’ev V.P., Carmona R., Domezain A., García-Gallego M., Hernando J.A., Rodríguez F., Ruiz-Rejón M. (2009). Mechanisms of polyploid evolution in fish: Polyploidy in Sturgeons. Biology, Conservation and Sustainable Development of Sturgeons.

[15] Trifonov A.V., Romanenko S.S., Beklemisheva R.V., Biltueva S.L., Makunin I.A., Lemskaya A.N., Kulemzina I.A., Stanyon R., Graphodatsky S.A. (2016). Evolutionary plasticity of acipenseriform genomes. Chromosoma.

[16] Kuhl H., Guiguen Y., Höhne C., Kreuz E., Du K., Klopp C., Lopez-Roques C., Yebra-Pimentel S.E., Ciorpac M., Gessner J. (2021). A 180 Myr-old female-specific genome region in sturgeon reveals the oldest known vertebrate sex determining system with undifferentiated sex chromosomes. Philos. Trans. R. Soc. B.

[17] Shelton L.W., Mims D.S. (2012). Evidence for female heterogametic sex determination in paddlefish *Polyodon spathula* based on gynogenesis. Aquaculture.

[18] Havelka M., Hulák M., Bailie D.A., Prodöhl P.A., Flajšhans M. (2013). Extensive Genome Duplication in Sturgeons: New Evidence from Microsatellite Data. J. Appl. Ichthyol..

[19] Káldy J., Mozsár A., Fazekas G., Farkas M., Fazekas D.L., Fazekas G.L., Goda K., Gyöngy Z., Kovács B., Semmens K. (2020). Hybridization of Russian Sturgeon (*Acipenser gueldenstaedtii*, Brandt and Ratzeberg, 1833) and American Paddlefish (*Polyodon spathula*, Walbaum 1792) and evaluation of their progeny. Genes.

[20] Flamio R., Chojnacki A.K., DeLonay J.A., Dodson J.M., Gocker M.R., Jenkins A.J., Powell J., Heist J.E. (2021). Production of haploid gynogens to inform genomic resource development in the paleotetraploid pallid sturgeon (*Scaphirhynchus albus*). Aquaculture.

[21] Káldy J., Fazekas G., Kovács B., Molnár M., Lázár B., Pálinkás-Bodzsár N., Ljubobratović U., Fazekas G., Kovács G., Várkonyi E. (2024). Unidirectional hybridization between American paddlefish *Polyodon spathula* (Walbaum, 1792) and sterlet *Acipenser ruthenus* (Linnaeus, 1758). PeerJ.

[22] Fontana F., Congiu L., Mudrak A.V., Quattro M.J., Smith I.J.T., Ware K., Doroshov I.S. (2008). Evidence of hexaploid karyotype in shortnose sturgeon. Genome.

[23] Bruslé J., Bruslé S. (1983). La gonadogenèse des Poissons. Reprod. Nutr. Dév..

[24] Arnold M.L., Hodges S.A. (1995). Are natural hybrids fit or unfit relative to their parents?. Trends Ecol. Evol..

[25] Chapman M.A., Burke J.M. (2007). Genetic divergence and hybrid speciation. Evolution.

[26] Vasil’ev V.P., Rachek E.I., Lebedeva E.B., Vasil’eva E.D. (2014). Karyological study in backcross hybrids between the sterlet, *Acipenser ruthenus*, and kaluga, *A. dauricus* (Actinopterygii: Acipenseriformes: Acipenseridae): *A. ruthenus* × (*A. ruthenus* × *A. dauricus*) and *A. dauricus* × (*A. ruthenus* × *A. dauricus*). Acta Ichthyol. Piscat..

[27] Linhartová Z., Havelka M., Pšenička M., Flajšhans M. (2018). Interspecific Hybridization of Sturgeon Species Affects Differently Their Gonadal Development. Czech J. Anim. Sci..

[28] Štěch L., Linhart O., Shelton L.W., Mims D.S. (1999). Minimally Invasive Surgical Removal of Ovulated Eggs from Paddlefish. Aquac. Int..

[29] Heist E.J., Nicholson E.H., Sipiorski J.T., Keeney D.B. (2002). Microsatellite Markers for the Paddlefish (*Polyodon spathula*). Conserv. Genet..

[30] McQuown E.C., Sloss B.L., Sheehan R.J., Rodzen J., Tranah G.J., May B. (2000). Microsatellite Analysis of Genetic Variation in Sturgeon: New Primer Sequences for *Scaphirhynchus* and *Acipenser*. Trans. Am. Fish. Soc..

[31] Ruan R., Feng T., Li Y., Yue H., Ye H., Du H., Liu Q., Ruan J., Li C., Wei Q. (2021). Screening and identification of female-specific DNA sequences in octaploid sturgeon using comparative genomics with high-throughput sequencing. Genomics.

[32] Hurvitz A., Jackson K., Degani G., Levavi-Sivan B. (2007). Use of endoscopy for gender and ovarian stage determinations in Russian sturgeon (*Acipenser gueldenstaedtii*) grown in aquaculture. Aquaculture.

[33] Vizziano-Cantonnet D., Di Landro S., Lasalle A., Martinez A., Mazzoni S.T., Quagio-Grassiotto I. (2016). Identification of the Molecular Sex-Differentiation Period in the Siberian Sturgeon. Mol. Reprod. Dev..

[34] Fajkowska M., Ostaszewska T., Rzepkowska M. (2019). Review: Molecular mechanisms of sex differentiation in sturgeons. Rev. Aquac..

[35] Devlin H.R., Nagahama Y. (2002). Sex determination and sex differentiation in fish: An overview of genetic, physiological, and environmental influences. Aquaculture.

[36] Guiguen Y., Fostier A., Piferrer F., Chang C.F. (2010). Ovarian aromatase and estrogens: A pivotal role for gonadal sex differentiation and sex change in fish. Gen. Comp. Endocrinol..

[37] Hagihara S., Yamashita R., Yamamoto S., Ishihara M., Abe T., Ijiri S., Adachi S. (2014). Identification of genes involved in gonadal sex differentiation and the dimorphic expression pattern in undifferentiated gonads of Russian sturgeon *Acipenser gueldenstaedtii* Brandt & Ratzeburg, 1833. J. Appl. Ichthyol..

[38] Wuertz S., Güralp H., Pšenička M., Chebanov M., Wang H.-P., Piferrer F., Chen S.-L., Shen Z.-G. (2019). Sex Determination in Sturgeon. Sex Control in Aquaculture.

[39] Omoto N., Maebayashi M., Adachi S., Arai K., Yamauchi K. (2005). Sex ratios of triploids and gynogenetic diploids induced in the hybrid sturgeon, the bester (*Huso huso* female × *Acipenser ruthenus* male). Aquaculture.

[40] Van Eenennaam A.L., Van Eenennaam J.P., Medrano J.F., Doroshov S.I. (1999). Evidence of female heterogametic genetic sex determination in white sturgeon. J. Hered..

[41] Wertheim B., Beukeboom L.W., van de Zande L. (2013). Polyploidy in animals: Effects of gene expression on sex determination, evolution and ecology. Cytogenet. Genome Res..

[42] Stöck M., Dedukh D., Reifová R., Lamatsch D.K., Starostová Z., Janko K. (2021). Sex chromosomes in meiotic, hemiclonal, clonal and polyploid hybrid vertebrates: Along the ‘extended speciation continuum’. Philos. Trans. R. Soc. B.

[43] Vasil’ev V.P., Rachek E.I., Amvrosov D.Y., Barmintseva A.E., Vasil’eva E.D. (2021). Fertility of females of sturgeon hybrids obtained from species with different levels of ploidy (*Acipenser ruthenus* and *A. dauricus*) and their cloning. J. Appl. Ichthyol..

[44] Haldane J. (1922). Sex ration and unisexual sterility in animal hybrids. J. Genet..

